# Prediction of Pulmonary Function Parameters Based on a Combination Algorithm

**DOI:** 10.3390/bioengineering9040136

**Published:** 2022-03-25

**Authors:** Ruishi Zhou, Peng Wang, Yueqi Li, Xiuying Mou, Zhan Zhao, Xianxiang Chen, Lidong Du, Ting Yang, Qingyuan Zhan, Zhen Fang

**Affiliations:** 1 Aerospace Information Research Institute, Chinese Academy of Sciences (AIRCAS), Beijing 100190, China; zhouruishi17@mails.ucas.ac.cn (R.Z.); wangpeng01@aircas.ac.cn (P.W.); liyueqi18@mails.ucas.ac.cn (Y.L.); mouxiuying18@mails.ucas.ac.cn (X.M.); zhaozhan@mail.ie.ac.cn (Z.Z.); chenxx@aircas.ac.cn (X.C.); 2 School of Electronic, Electrical and Communication Engineering, University of Chinese Academy of Sciences, Beijing 100190, China; 3 Personalized Management of Chronic Respiratory Disease, Chinese Academy of Medical Sciences, Beijing 100190, China; 4 Department of Respiratory Medicine, China-Japan Friendship Hospital, Beijing 100029, China

**Keywords:** combination algorithm, support vector machines, extreme gradient boosting, one-dimensional convolutional neural network, improved K-nearest neighbor

## Abstract

Objective: Pulmonary function parameters play a pivotal role in the assessment of respiratory diseases. However, the accuracy of the existing methods for the prediction of pulmonary function parameters is low. This study proposes a combination algorithm to improve the accuracy of pulmonary function parameter prediction. Methods: We first established a system to collect volumetric capnography and then processed the data with a combination algorithm to predict pulmonary function parameters. The algorithm consists of three main parts: a medical feature regression structure consisting of support vector machines (SVM) and extreme gradient boosting (XGBoost) algorithms, a sequence feature regression structure consisting of one-dimensional convolutional neural network (1D-CNN), and an error correction structure using improved K-nearest neighbor (KNN) algorithm. Results: The root mean square error (RMSE) of the pulmonary function parameters predicted by the combination algorithm was less than 0.39L and the R^2^ was found to be greater than 0.85 through a ten-fold cross-validation experiment. Conclusion: Compared with the existing methods for predicting pulmonary function parameters, the present algorithm can achieve a higher accuracy rate. At the same time, this algorithm uses specific processing structures for different features, and the interpretability of the algorithm is ensured while mining the feature depth information.

## 1. Introduction

In recent years, artificial intelligence (AI) and machine learning (ML) have rapidly evolved in various fields, including healthcare. These methods can help detect diseases, improve pathological classification, and predict disease patterns and epidemiology, a prime example of which is ML-based algorithms developed during the COVID-19 pandemic [1,2]. In addition, the authors of [3] created a system that developed and trained a neural network model for the diagnosis of diabetes mellitus in pregnant women and the accuracy of the trained network was over 92%. In [4], a fuzzy expert system was proposed for diagnosing and analyzing human diseases. The system not only indicates if the disease is present but also indicates the level at which the disease is present. It is notable that this approach for diagnosing human diseases has an accuracy and reliability of 97%. The authors of [5], developed an expert system for oral ulcers that focuses on four common oral ulcers. In addition, the study of medical image data in [6], used CT images for the segmentation and classification of small hepatocellular carcinoma, and achieved good results. The accuracy of the segmentation was 0.9049, and the accuracy of the classification was 0.838.

The above studies are all able to achieve good results in their corresponding fields, but not in the field of chronic respiratory diseases.

At the same time, chronic respiratory diseases, including chronic obstructive pulmonary disease (COPD) and asthma, are significantly increasing in regard to morbidity and mortality worldwide. They can affect individuals of all age groups and cause over 3 million deaths each year according to World Health Organization data [7,8]. Therefore, it is important to apply AI technology to the assessment, diagnosis, and treatment stages of chronic respiratory diseases, thereby reducing patient morbidity and mortality.

The assessment of the patient’s pulmonary function parameters is a prerequisite for the prevention and treatment of chronic respiratory diseases. Today, spirometry is one of the most widely-used techniques to assess pulmonary function [9]. Unfortunately, spirometry has strict end-of-test criteria and poor patient compliance leads to the low accuracy of pulmonary function parameters. Schermer et al. found that 50% of the spirometry methods were inaccurate in terms of pulmonary function parameters [10]. For this reason, many scholars have conducted studies on the prediction of lung function parameters. Sharan et al. investigated the prediction of lung function parameters using coughing sounds [11], while Ioachimescu et al. (2020) performed partial lung function prediction based on age, sex, race, height, and weight and using an artificial neural network (ANN) algorithm [12]. Miyoshi et al. (2020) developed regression equations to estimate forced vital capacity (FVC) and forced expiratory volume in one second (FEV1) [13]. Chen et al. developed an FEV1 and FVC prediction model based on multi-output support vector regression [14].

Meanwhile, volumetric capnography has emerged as a technique for pulmonary function assessment that helps to solve the problem of inaccurate prediction of pulmonary function parameters and has wide application prospects [15]. Jarenbäck et al. (2018) obtained an index of efficiency of tidal ventilation with respect to CO_2_ exchange (efficiency index, EFFi) in volumetric capnography and tested the hypothesis that EFFi may be used for the diagnostics and grading of COPD [16]. Kellerer et al. (2020) conducted a systematic analysis of the relationship between capnovolumetric and conventional lung function parameters to help in the interpretation of capnovolumetric parameters [17]. Although these authors conducted preliminary studies on volumetric capnography, they did not use volumetric capnography data for the specific prediction of pulmonary function parameters. They only elaborated on the correlation between volumetric capnography and pulmonary function parameters such as FVC and FEV1.

Therefore, in this paper, a combination algorithm based on volumetric capnography data is proposed for the first time to solve the problem of the accuracy of pulmonary function parameters prediction, thus improving the accuracy of pulmonary function parameter prediction. 

The novelty and contributions of this study are as follows.

(1) This paper is the first to propose the use of volumetric capnography data for the prediction of pulmonary function parameters, which is more accessible and less demanding for testers than other studies.
(2) The algorithm proposed in this paper combines the advantages of traditional machine learning algorithms for processing high-dimensional medical features and deep learning for learning low-dimensional sequence features, to improve the accuracy of pulmonary function parameter prediction.
(3) This paper provides a reference paradigm for other medical data processing by handling high-dimensional features and low-dimensional features in medical data.

In the subsequent sections, we first establish the signal acquisition system and compensate the system signal using an adaptive control algorithm. Then, the proposed combination algorithm is described in detail and the performance of different algorithms is compared. The experimental results show that the combination algorithm proposed in this paper has high accuracy in the prediction of pulmonary function parameters and can significantly improve the quality of pulmonary function assessment.

## 2. Materials and Methods

### 2.1. Signal Acquisition System

**Figure 1:**
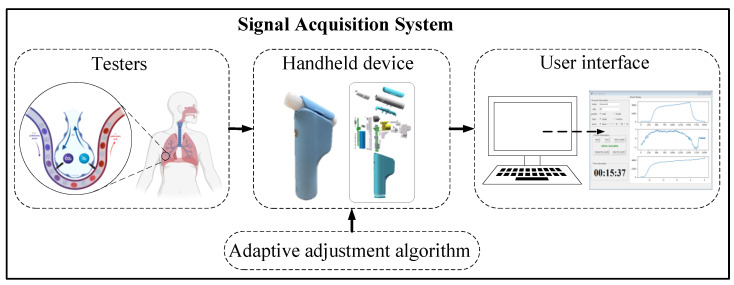
Signal acquisition system.

To enable better volumetric capnography data acquisition, we built a homemade signal acquisition system. We tested the performance of the system with a PWG-33 pulmonary waveform generator and a carbon dioxide concentration verification platform, and the system’s measurement error was within 5%. The signal acquisition system is shown in Figure 1. It mainly contains handheld multi-sensor devices and a user interface, and the system signal is compensated by an adaptive adjustment algorithm.

#### 2.1.1. Devices and User Interface

**Figure 2:**
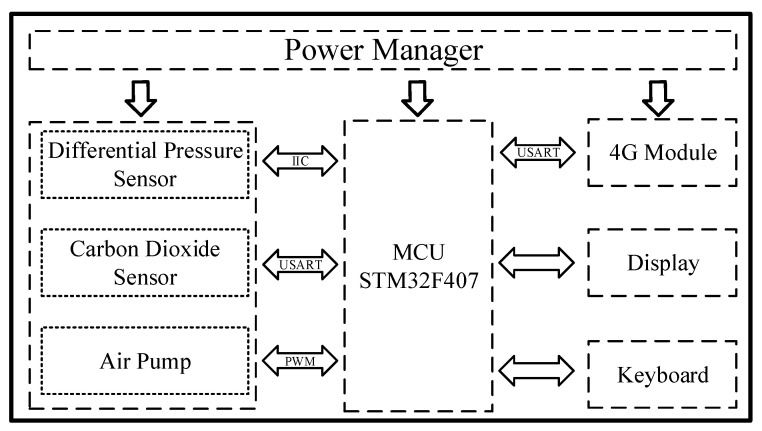
Embedded system of the handheld device. The microcontroller unit (MCU) communicates with the differential pressure sensor via Inter-Integrated Circuit (IIC) protocol, with the carbon dioxide sensor via Universal Synchronous/Asynchronous Receiver/Transmitter (USART) protocol, and the air pump control via pulse-width modulation (PWM) wave.

The embedded system of the handheld device is shown in Figure 2, including a microprocessor module, power management module, 4G communication module, display module, keyboard control module, a sensor acquisition module [18]. 

The microprocessor module is used to perform adaptive control algorithm and data processing, the power management module is responsible for the power supply of the entire device, the 4G communication module is responsible for signal transmission, the display module and the keyboard control module are used to interact with the user, and the sensor acquisition module is used for data acquisition.

The user interface mainly includes a personal information window, a control information window, a time information window, and a result display window. Firstly, the user fills in the contents of the personal information window as prompted. Then, different buttons are selected for interaction in the control information window. When the start button is clicked, the user interface plays a guided breathing tone and collects the user’s breathing information. At the same time, the time information window displays and records the breath time information. After breathing, the user can click the show button to display the results. The respiratory flow rate, the respiratory carbon dioxide concentration, and the volumetric capnography will be displayed in the result display window. Finally, the user can click the save results button to save the collected information in CSV format locally for subsequent processing and analysis.

#### 2.1.2. Adaptive Adjustment Algorithm

During respiration, the flow rate will always change. As a result, inconsistencies in the characteristic parameters of volumetric capnography can occur under different respiratory conditions. Therefore, the signal acquisition system uses an adaptive adjustment algorithm based on minimum prediction error to ensure the accuracy of volumetric capnography measurements. The algorithm can ensure the consistency of the characteristic parameters of volumetric capnography, and control the flow in advance to reduce the lag in the flow control due to the ability to predict the flow according to the actual flow. The algorithm is similar to the idea of minimizing the local structure error [19,20,21].

The adaptive adjustment algorithm is mainly divided into three stages, the prediction of respiratory flow at the next moment, the calculation of forecast error, and the adaptive adjustment of smoothing parameters.

- Prediction of respiratory flow at the next moment.

Set the initial sampling flow $f_{0}$, the initial smoothing parameter $\alpha_{0}$ and the prediction window size N. Get the actual respiratory flow $F_{i-N+1},F_{i-N+2},\ldots \ldots F_{i-1},F_{i}$ at the previous N moments through the sampling of the differential pressure sensor. According to the traditional exponential smoothing algorithm, predict the respiratory flow at the next moment ${\hat{F}}_{i+1}:$

(1) $${\hat{F}}_{i+1}=\alpha_{0}F_{i}+\alpha_{0}\left(1-\alpha_{0}\right)F_{i-1}+\alpha_{0}{\left(1-\alpha_{0}\right)}^{2}F_{i-2}+\ldots$$

- Calculation of forecast error.

Get the actual respiratory flow $F_{i+1}$ at time i+1, and record the difference in flow $E_{i+1}$ between the predicted flow ${\hat{F}}_{i+1}$ at i+1 time and the actual respiratory flow $F_{i+1}:$

(2) $$E_{i+1}={\hat{F}}_{i+1}-F_{i+1}$$

Set the error calculation window size W, calculate the mean value of the difference in the sliding window $\bar{E_{w}}$:

(3) $$\bar{E_{w}}=\overset{i}{\underset{x=i-w}{\sum }}\frac{E_{x}}{W}$$

- Adaptive adjustment of smoothing parameters.

According to $E_{i+1},\bar{\;E_{w}}$, the self-adjustment coefficient β, the smoothing parameter α, adjust the smoothing parameter α:

(4) $$\alpha_{i+1}=\alpha_{i}\left(1+\beta \left(\frac{E_{i+1}}{\bar{E_{w}}}-1\right)\right)$$

Control the sampling flow $f_{i+1}$ according to the updated smoothing parameter $\alpha_{i+1}$:

(5) $$f_{i+1}=\alpha_{i+1}f_{i}+\alpha_{i+1}\left(1-\alpha_{i+1}\right)f_{i-1}+\alpha_{i+1}{\left(1-\alpha_{i+1}\right)}^{2}f_{i-2}+\ldots$$

In different breathing situations, the adaptive algorithm can adjust the sampling flow rate, solve the problem of inconsistencies in the characteristic parameters of the volumetric capnography under different respiratory flows, and ensure the accuracy of the volumetric capnography [22]. The details of the adaptive adjustment algorithm are shown in Algorithm 1.
**Algorithm 1:** Adaptive Adjustment Algorithm.**Input:**initial sampling flow $f_{0}$, initial smoothing parameter $\alpha_{0}$, the prediction window size N,
error calculation window size W, the self-adjustment coefficient β.**Output:**smoothing parameters $\alpha_{i+1}$, sampling flow $f_{i+1}$**while** obtaining the actual respiratory $F_{i}$
**do**
**for len**(F) < N
**do**

F = $F.add(F_{i}$)
predict the respiratory flow at the next moment ${\hat{F}}_{i+1}$
${\hat{F}}_{i+1}=\alpha_{0}F_{i}+\alpha_{0}\left(1-\alpha_{0}\right)F_{i-1}+\alpha_{0}{\left(1-\alpha_{0}\right)}^{2}F_{i-2}+\ldots$
obtain actual breathing flow at the *i* + 1 time point $F_{i+1}$
calculation of forecast error
$E_{i+1}={\hat{F}}_{i+1}-F_{i+1}$
calculate the mean value of the difference in the sliding window $\bar{E_{w}}$
$\bar{E_{w}}=\overset{i}{\underset{x=i-w}{\sum }}\frac{E_{x}}{W}$
adaptive adjustment of smoothing parameters $\alpha_{i+1}$ and sampling flow $f_{i+1}$
$\alpha_{i+1}=\alpha_{i}\left(1+\beta \left(\frac{E_{i+1}}{\bar{E_{w}}}-1\right)\right)$
$f_{i+1}=\alpha_{i+1}f_{i}+\alpha_{i+1}\left(1-\alpha_{i+1}\right)f_{i-1}+\alpha_{i+1}{\left(1-\alpha_{i+1}\right)}^{2}f_{i-2}+\ldots$**end**

### 2.2. Combination Algorithm

For the traditional single-structure pulmonary function regression algorithm, the mining of data information is limited by the structural design. Traditional machine learning algorithms can mine relationships in high-dimensional data (medical features, etc.) very well and provide good explanations, but the accuracy needs to be improved [23]. Deep learning algorithms can mine deep relationships from low-dimensional data (sequence data), but cannot provide good explanations [24].

Inspired by the combinatorial structure [25], we propose the combination algorithm, which mainly consists of three parts: a medical feature regression structure, a sequence feature regression structure, and an error correction structure. The medical features are processed by the traditional machine learning algorithm and the sequence data are processed by the deep learning algorithm. Finally, the results of the two are effectively combined to improve the accuracy rate. The algorithm ensures both a good interpretation of high-dimensional medical features and the full utilization of low-dimensional sequence data.

#### 2.2.1. Medical Feature Regression Structure

We constructed a two-layer medical feature regression structure using support vector machines (SVM) and extreme gradient boosting (XGBoost) algorithms to take full advantage of the medical data in the volumetric capnography and also based on the a priori knowledge of the airflow limitation cutoffs of GOLD2020 [26].

For the prior medical knowledge of the airflow limitation cutoff point in GOLD2020, we first need to build a first-level classification task to determine whether a patient has airflow limitation or not. For this classification task, the input features are high-dimensional data such as medical features and demographic features, for which the support vector machine (SVM) algorithm has a good processing effect. SVM is an optimal margin-based classification technique in machine learning [27].

Since the SVM algorithm is used for the binary classification task, the results obtained are too sparse with only two possibilities. Therefore, to provide more information for the secondary prediction, we used the sigmoid function to make the output result of SVM probabilistic, which contains more information and is denser. The Sigmoid function is calculated as:

(6) $$\sigma \left(z\right)=\frac{1}{1+e^{-z}}$$

We combined the output probability values of the SVM with the original features as the input features for the second-level prediction to perform the regression prediction of the FEV1 and FVC parameters. For the regression prediction task, we chose the XGBoost algorithm for secondary regression in order to show the importance and interpretability of each feature on the regression results.

XGBoost was recently proposed by Chen and Guestrinis [28]. It is based on the original framework of gradient boosting, for a given data set with n examples and m features $D=\left\{\left(x_{i},y_{i}\right)\right\}\left(\left|D\right|=n,x_{i}\in {\mathbb{R}}^{m},y_{i}\in \mathbb{R}\right)$, and uses K additive trees to approximate the output ${\hat{y}}_{i}$ as the following:

(7) $${\hat{y}}_{i}=\phi \left(x_{i}\right)=\overset{K}{\underset{k=1}{\sum }}f_{k}\left(x_{i}\right),f_{k}\in ℱ$$

where $f_{k}$ is an independent classification and regression tree (CART) at each of the k steps, which map the input variables $x_{i}$ to $y_{i}$. $ℱ=\left\{f\left(x\right)=w_{q\left(x\right)}\right\}\left(q:{\mathbb{R}}^{m}\to T,w\in {\mathbb{R}}^{T}\right)$ is the space of all regression trees.

To learn the set of functions used in the model, we minimized the following regularized objective.

(8) $$ℒ\left(\phi \right)=\underset{i}{\sum }l\left({\hat{y}}_{i},y_{i}\right)+\underset{k}{\sum }\Omega \left(f_{k}\right)$$

(9) $$\mathrm{where}\;\Omega \left(f\right)=\gamma T+\frac{1}{2}\lambda ∥w{∥}^{2}$$

The training loss function l and the regularization term Ω make up the regularized objective function. The difference between the predicted value ${\hat{y}}_{i}$ and the value $y_{i}$ is measured by the training loss function l. The regularization term Ω assesses the model’s complexity and helps to smooth the final learned weight to avoid overfitting.

In addition, XGBoost includes two key techniques: shrinkage and column subsampling. At each stage of boosting, the shrinkage algorithm scales the newly supplied weights, reducing the effect of each tree and overfitting. To speed up the training process, column subsampling only selects a random subset of input characteristics while creating a tree [29].

**Figure 3:**
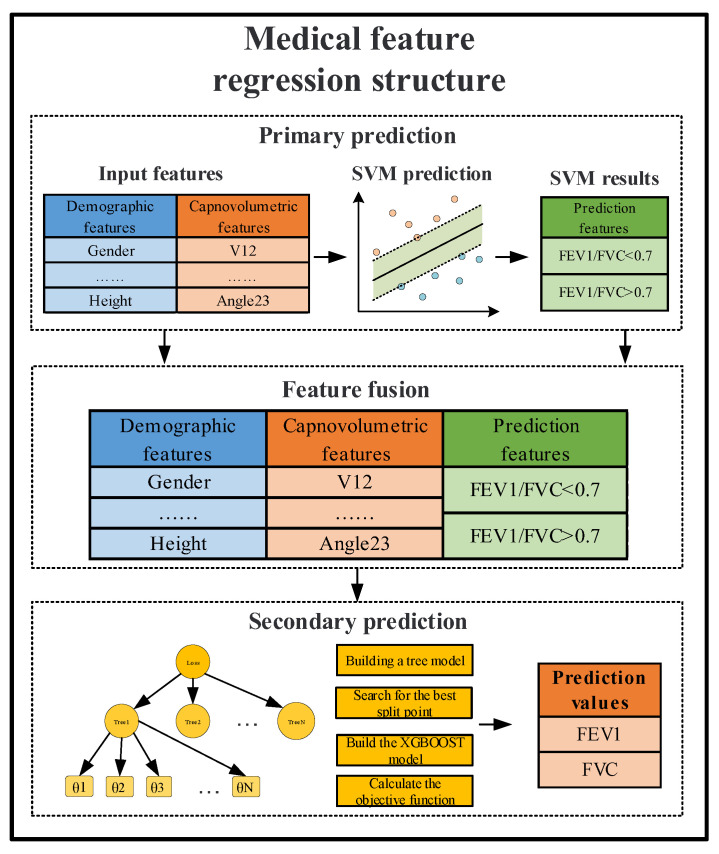
Structural flow chart of the medical feature regression structure.

The medical feature regression structure is shown in Figure 3.

First, the first-level prediction structure was constructed using SVM with FEV1/FVC = 0.7 as the threshold to classify and predict the airway obstruction condition and obtain the corresponding probability values [30]. Subsequently, the probability results of the first-level prediction were combined with the original feature as the input features of the second-level prediction structure. XGBoost was used to construct the secondary prediction structure, and the prediction results of the pulmonary function parameters were then output. During the implementation of the algorithm, we used a heuristic search for the selection of hyperparameters to achieve optimal results [31].

#### 2.2.2. Sequence Feature Regression Structure

To be able to make full use of the volumetric capnography data, we want to mine information from the low-dimensional raw sequence signals, in addition to using traditional machine learning algorithms for the high-dimensional medical and demographic features.

For the original low-dimensional CO_2_ sequence data, deep learning networks have better learning abilities. When choosing the deep learning network structure, we considered that the original sequence information is only one-dimensional in depth; therefore, if we use network structures such as long short-term memory (LSTM) and gated recurrent units (GRU), it will increase the computational effort when performing the data processing with no effective improvement. Therefore, we chose the simpler one-dimensional convolutional neural network (1D-CNN), which is widely used in medical sequence signals [32,33,34].

Given a sequence of CO_2_, $C_{1:n}=C_{1},\ldots ,C_{n}$, a 1D convolution of width-k is the result of moving a sliding window of size s over the sequence, and applying the same convolution filter or kernel to each window in the sequence, i.e., a dot-product between the concatenation of the vectors in a given window and a weight vector u, which is then often followed by a non-linear activation function g. We chose the rectified linear unit (ReLU) function as the activation function to ensure the updating ability of the network when performing gradient backpropagation.

(10) g(x)=max(0,x)

The convolution filter is applied to each window, resulting in scalar values $r_{i}$, each for the i window:

(11) $$r_{i}=g\left(x_{i}\cdot u\right)\in R$$

In practice, one typically applies more filters, $u_{1},u_{2}\ldots ,u_{l}$, which can then be represented as a vector multiplied by a matrix U and with the addition of a bias term b:

(12) $$r_{i}=g\left(x_{i}\cdot U+b\right)$$

**Figure 4:**
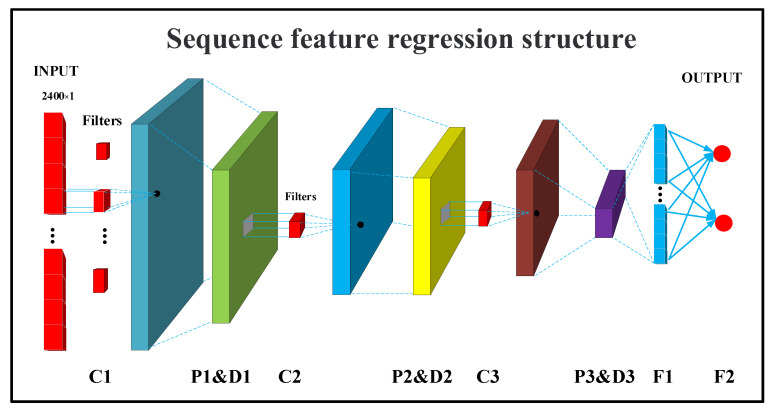
Structural flow chart of the sequence feature regression structure.

The network structure is shown in Figure 4.

The network parameters are shown in Table 1.

When building the network, we followed the general structure including a convolution layer, pooling layer and a dropout layer [35]. The convolution layer can be used to extract features from the sequence information, the pooling layer can be used to reduce the training parameters, and the dropout layer can be used to avoid training overfitting. We stacked the generic structure three times to ensure that the output vector has a larger receptive field [36]. After the stacked structure, we added a flattened layer to flatten the vector. Finally, we added a fully connected layer to map the flattened vectors to the FEV1 and FVC parameters and chose the mean square error as the loss function. When choosing the size and number of convolutional kernels, we chose a 1 × 5 convolutional kernel size, considering that a smaller convolutional kernel filter can help to improve computational efficiency and extract clearer features [37]. The number of convolutional kernels is generally a power of 2. In this case, we chose 32, 64, and 32 convolutional kernels, respectively.

#### 2.2.3. Error Correction Structure

After processing high-dimensional information by traditional machine learning algorithms and low-dimensional information by deep learning algorithms, we need to combine the results of both algorithms organically to combine the respective advantages of both algorithms. To ensure the operability and interpretability of the synthesis results, we chose the improved K-nearest neighbor (KNN) regression algorithm as the output of the final results [38,39].

The traditional KNN algorithm is mainly used for classification problems. For two points $x=\left(x_{1},x_{2},\ldots ,x_{n}\right)$ and $y=\left(y_{1},y_{2},\ldots ,y_{n}\right)$ on an n-dimensional real vector space $R^{n}$, we can define a more generalized distance $L_{p}$ between the two points, that is, the Minkowski distance as

(13) $$L_{p}\left(x,y\right)={\left(\overset{n}{\underset{i=1}{\sum }}{\left|x_{i}-y_{i}\right|}^{p}\right)}^{\frac{1}{p}}$$

Here, we used the spatial distance when p=2, i.e., the Euclidean distance.

(14) $$L_{2}\left(x,y\right)=\sqrt{\overset{n}{\underset{i=1}{\sum }}{\left(x_{i}-y_{i}\right)}^{2}}$$

The traditional KNN algorithm divides the test samples into classes of the k-nearest samples in the n-dimensional space. We made certain improvements to the KNN algorithm to fit the regression problem here.

For any training sample, with the medical feature regression structure and the sequences feature regression structure, we get the output of a four-dimensional vector

(15) $$Train\_x_{i}=\left(FEV1\_med_{i},FVC\_med_{i},FEV1\_seq_{i},FVC\_seq_{i}\right)$$

The true output of that sample is

(16) $$Train\_y_{i}=\left(FEV1_{i},FVC_{i}\right)$$

For the test sample, the same four-dimensional output can be obtained with the two structures mentioned above.

(17) $$Test\_x_{i}=\left(FEV1\_med_{i},FVC\_med_{i},FEV1\_seq_{i},FVC\_seq_{i}\right)$$

In the four-dimensional space, we calculated the distance between the test sample and all training samples. We chose the K closest distance training samples and took the true output of those K training samples and we calculated the mean of their pulmonary function parameters as the final output of our test samples.

(18) $$Test\_y_{i}=\left(\frac{1}{k}\overset{K}{\underset{i=1}{\sum }}FEV1_{i},\frac{1}{k}\overset{K}{\underset{i=1}{\sum }}FVC_{i}\right)$$

With the improved KNN algorithm, we fully consider the output predicted by each traditional machine learning algorithm and deep learning algorithm, then integrate the results to arrive at the final prediction result. The improved KNN algorithm has advantages such as simple computation and strong interpretation.

The details of the combination algorithm flowchart are shown in Algorithm 2.
**Algorithm 2:** Combination Algorithm.**Input:**Test set $X_{test}={\left\{x_{med}{}^{\left(i\right)},x_{seq}{}^{\left(i\right)}\right\}}_{i=1,\ldots ,n}$,$\;x_{med}{}^{\left(i\right)}$ is a medical feature vector, $x_{seq}{}^{\left(i\right)}$ is the sequence feature vector**Output:**$Y_{test}={\left\{y^{\left(i\right)}\right\}}_{i=1,\ldots ,n}$,$\;y^{\left(i\right)}$ is the pulmonary function parameter vector**for** i <= n
**do**
Medical Feature Regression Structure

 $x_{med}{}^{\left(i\right)}$ through SVM model to obtain $y_{SVM}^{\left(i\right)}$

 Fusion of features from $x_{med}{}^{\left(i\right)}$ and $y_{SVM}^{\left(i\right)}$ to obtain $x_{xgboost}{}^{\left(i\right)}$

 $x_{xgboost}{}^{\left(i\right)}$ through XGBoost model to obtain $y_{med}^{\left(i\right)}$
Sequence feature regression structure

 $x_{seq}{}^{\left(i\right)}$ through 1D-CNN model to obtain $y_{seq}^{\left(i\right)}$

Error correction structure

 By splicing the vectors $y_{med}^{\left(i\right)}$ and $y_{seq}^{\left(i\right)}$, we obtain the vector $x_{KNN}{}^{\left(i\right)}$


 $x_{KNN}{}^{\left(i\right)}$ through the KNN model to obtain $y^{\left(i\right)}$
**end**

## 3. Results

### 3.1. Regression Evaluation Index

Given the sensitivity of medical data to maximum error, we propose a comprehensive error evaluation index comprehensive percentage error (CPE), which integrates the maximum percentage error, mean absolute percentage error, and root mean square percentage error and is more suitable for the prediction evaluation of medical data [40].

The maximum percentage error (MPE) computes the maximum residual error percentage, a metric that captures the worst-case error between the predicted value and the true value. The mean absolute percentage error (MAPE) is an evaluation metric for regression problems. This metric is sensitive to relative errors. It is for example not changed by a global scaling of the target variable. The root mean square percentage error (RMSPE) is a measure of the deviation between the predicted value and the true value. They are defined as follows:

(19) $$\mathrm{MPE}\left(x_{i},y_{i}\right)=\max\left(\frac{\left|x_{i}-y_{i}\right|}{y_{i}}\right)\times 100$$

(20) $$\mathrm{MAPE}\left(x_{i},y_{i}\right)=\frac{1}{n}\overset{n}{\underset{i=1}{\sum }}\frac{\left|x_{i}-y_{i}\right|}{\max\left(\epsilon ,\left|y_{i}\right|\right)}\times 100$$

(21) $$\mathrm{RMSPE}\left(x_{i},y_{i}\right)=\frac{\sqrt{\frac{1}{n}\sum_{i=1}^{n}{\left(x_{i}-y_{i}\right)}^{2}}}{\bar{y_{i}}}\times 100$$

where $x_{i}$ is the predicted value, $y_{i}$ is the true value, n is the number of samples, $\bar{y_{i}}$ is the mean of the true values, and ϵ is an arbitrary small, yet strictly positive number to avoid undefined results when $y_{i}$ is zero.

The comprehensive percentage error (CPE), which is a combination of the maximum percentage error, mean absolute percentage error, and relative standard deviation, reflects the overall error of the regression results. The smaller the comprehensive error percentage, the better the regression results.

(22) $$\mathrm{CPE}\left(x_{i},y_{i}\right)=\frac{1}{3}\times \left(\mathrm{MPE}\left(x_{i},y_{i}\right)+\mathrm{MAPE}\left(x_{i},y_{i}\right)+\mathrm{RMSPE}\left(x_{i},y_{i}\right)\right)$$

Therefore, the accuracy rate (ACC) considering the combined comprehensive percentage error is

(23) $$\mathrm{ACC}\left(x_{i},y_{i}\right)=1-\mathrm{CPE}\left(x_{i},y_{i}\right)$$

### 3.2. Datasets

We performed volumetric capnography experiments and spirometry experiments on 1007 subjects (472 females and 535 males, aged 17–70 years). The sampling frequency of the volumetric capnography was 200 Hz, the sampling time of the data was greater than 20 s, and the length of the sequence of CO_2_ acquisition was greater than 4000 sampling points. Pulmonary function experiments were performed to obtain the pulmonary function parameters, FEV1 and FVC. A total of more than 1007 subjects were obtained with three types of characteristic data. The volumetric capnography features are described in Table 2. The statistical analysis table is shown in Table 3.

**Figure 5:**
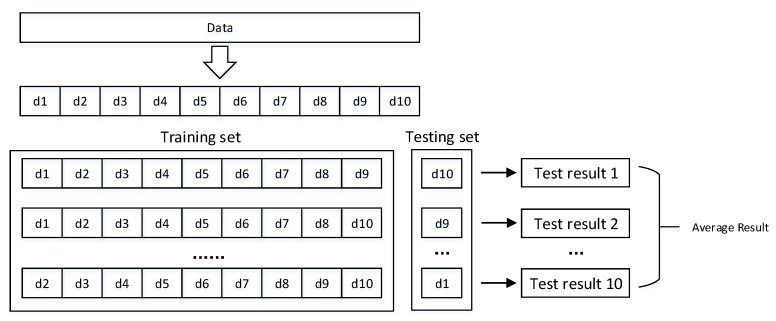
Schematic of 10-fold cross-validation method repeated 10 times.

To improve contrast and produce a balanced database, a 10-fold cross-validation strategy was applied 10 times to decrease generalization error in the training set. Figure 5 depicts the schematic diagram of the 10-fold cross-validation.

The dataset is first divided into 10 equally-sized, mutually-exclusive subsets: $\mathrm{Data}=d1\cup^{\;}d2\cup^{\;}\ldots \cup^{\;}d10$. $\mathrm{di}\cap^{\;}\mathrm{dj}$ is empty. Each subsection maintains the consistency of the data distribution, which is obtained through hierarchical sampling from the data. d1, d2…d10 is used as the test set to obtain 10 test results, and the average value of the 10 test results is used as a cross-validation result.

In this study, cross-validation was performed ten times, with the results of the ten cross-validations being averaged as the final result to assess the algorithm’s performance.

### 3.3. Results of the Algorithm

#### 3.3.1. Single-Structure Algorithm Results

To further assess the performance of our proposed combined algorithm, we compared single-structure machine learning and deep learning algorithms for pulmonary function prediction.

We used a single-structure conventional machine learning algorithm for pulmonary function prediction. Demographic and medical features were processed by SVM and XGBoost algorithms, and the results were evaluated using the relevant regression evaluation metrics.

The results of the conventional machine learning algorithm are shown in Table 4. For different pulmonary function parameters, FVC was a better predictor than FEV1 for R^2^ and ACC metrics. This is consistent with the medical phenomenon whereby FEV1 measurements are dependent on the effort of the tester and have poor predictive accuracy. In the RMSE index, FVC is 0.48 L greater than FEV1’s 0.43 L, and since it is an absolute value, FVC is greater than FEV1, making the absolute value of RMSE of FVC greater than FEV1. Overall, the traditional machine learning algorithm was able to do a better job of processing the medical features and getting the expected results. The maximum in the R^2^ metric was 0.79 and the maximum ACC was up to 79%. The result, however, still has room for improvement.

**Figure 6:**
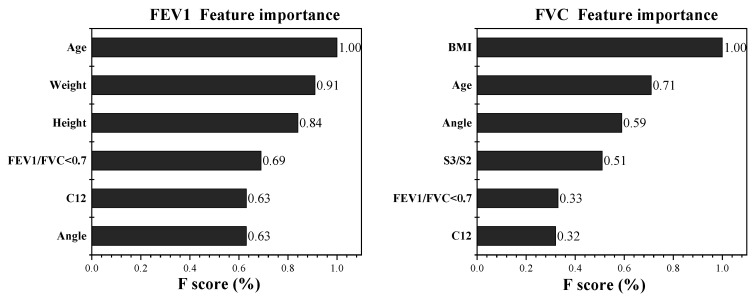
The features’ importance in conventional machine learning algorithms.

The features’ importance is shown in Figure 6. We can see that for the regression prediction of FEV1 versus FVC, the ranking of feature importance differs between the two. However, the two most important characteristics of both are demographic features, which is consistent with reality. As age and body size change, the human pulmonary function also undergoes significant changes.

**Figure 7:**
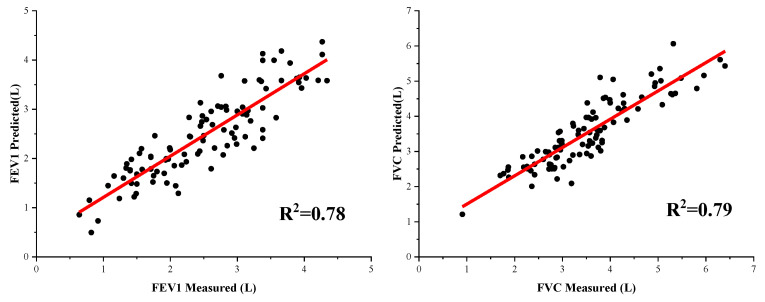
The fitting curves of the conventional machine learning results.

**Figure 8:**
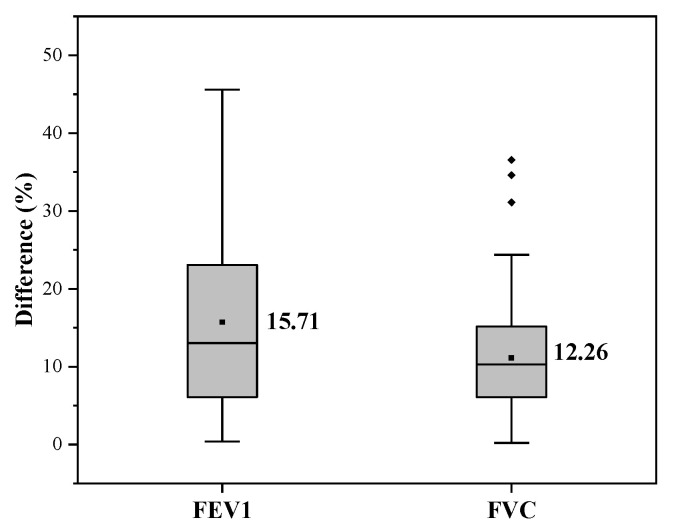
Box plot of error percentages of conventional machine learning.

The fitting curves and error percentages of conventional machine learning algorithms are shown in Figure 7 and Figure 8. Figure 8 shows that the average error of FEV1 prediction is 15.71%, and the average error of FVC is only 12.26%. Additionally, the median error for both is less than their mean error, indicating that the pulmonary function prediction results are acceptable for most testers. However, we also see that there are individual outliers in the results predicted by FVC, which indicates that the performance of FVC prediction is poor for a small number of results and there is still room for optimization.

We used a single-structured deep learning algorithm for pulmonary function prediction. The CO_2_ sequences are processed by a one-dimensional convolutional neural network and the results are evaluated using relevant evaluation metrics.

The results of the deep learning algorithm are shown in Table 5. In all evaluation metrics, the prediction of FVC is better than FEV1. Especially in the RMSE metric, FVC is 0.61 L, less than the 0.66 L of FEV1, which is different from common medical knowledge. Because the deep learning algorithm is end-to-end learning, which only mines the raw sequence data for regression and does not provide any medical prior knowledge, there may be results that contradict prior medical knowledge. This result shows that FVC is better than FEV1 in terms of raw sequence distribution, so all the results obtained by regression with a deep learning algorithm are better than FEV1, and probably since deep learning algorithms require larger datasets, they do not show good performance for the dataset used in this paper.

**Figure 9:**
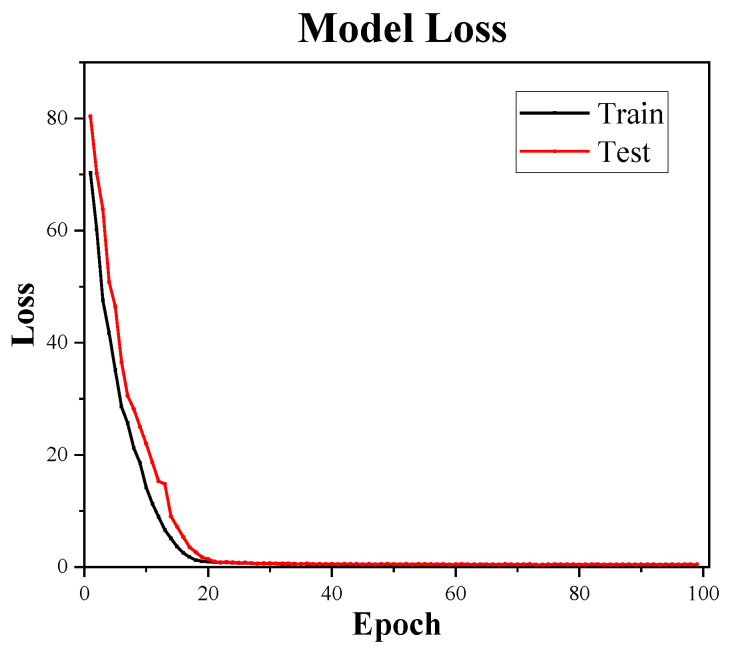
Training curves of the 1D-CNN network.

Figure 9 shows the training curves of the 1D-CNN network. We use a learning rate that is initially 0.03 and decays as the epoch increases. As can be seen from Figure 9, the network is trained normally and the model loss gradually decreases within 100 epochs without any phenomenon such as overfitting.

**Figure 10:**
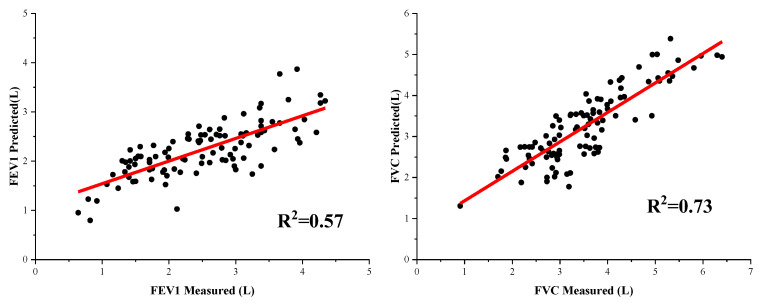
The fitting curves of the deep learning results.

**Figure 11:**
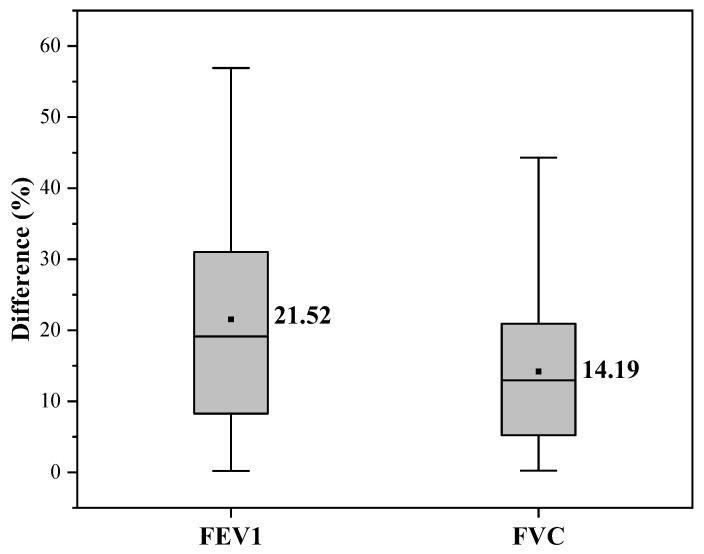
Box plot of error percentages of deep learning.

The fitting curves and error percentages of the deep learning algorithm results are shown in Figure 10 and Figure 11. As can be seen in Figure 10, the prediction performance of FEV1 needs to be improved, with an R^2^ of 0.57. Figure 11 shows that the average error of FEV1 prediction is 21.52%, and the average error of FVC is 14.19%. As can be seen from the figure, there are no outliers in the predicted results using the deep learning algorithm, which indicates that the deep learning algorithm can tap into the depth of information in the data and fit all the data distributions as much as possible, but its prediction performance needs to be improved.

#### 3.3.2. Combination Algorithm Results

We used combination algorithms to regress medical features using traditional machine learning algorithms and sequence features using deep learning algorithms, and finally, we combined the results through an error correction structure to perform a quadratic regression.

Table 6 shows the results of the combination algorithm. On the ACC index, both FEV1 and FVC were able to reach 80% and above, and the prediction of FVC was 85%, which is a good result. On the R^2^ index, both FEV1 and FVC reached 0.85 and above. This indicates a good fit of the results. On the RMSE metric, the FVC prediction result of 0.39 is greater than that of 0.35 for FEV1, which may be because the combined algorithm combines the output of traditional machine learning algorithms and includes prior medical knowledge in the execution process.

**Figure 12:**
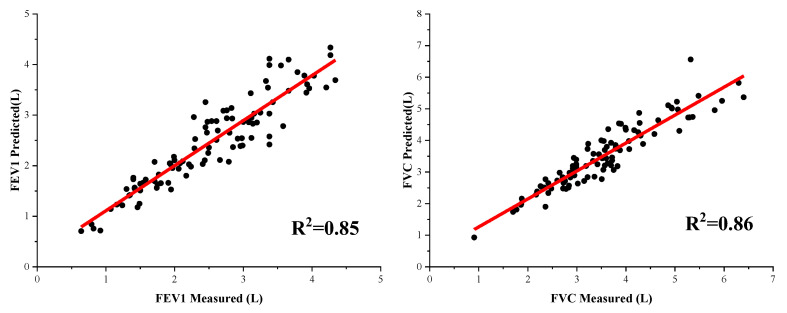
The fitting curves of the combination algorithm results.

**Figure 13:**
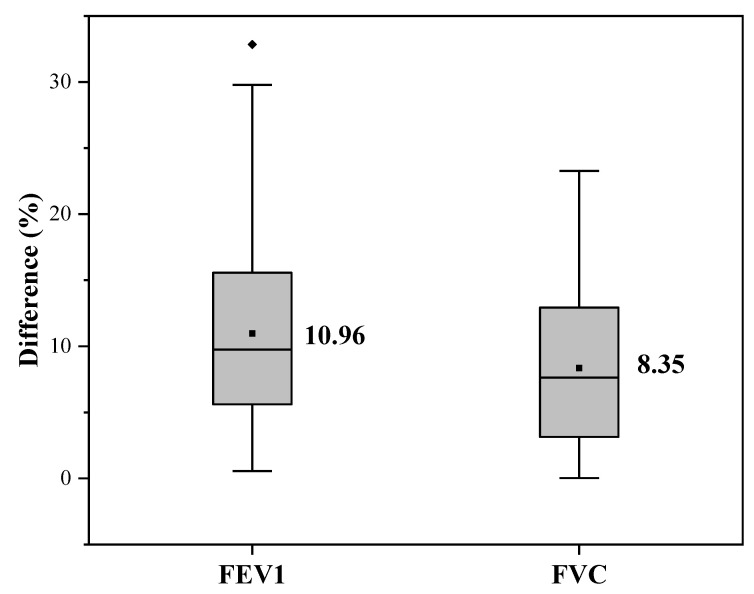
Box plot of error percentages of combination algorithm.

The fitting curves and error percentage of the combination algorithm’s results are shown in Figure 12 and Figure 13. As can be seen from Figure 13, the average errors of both FEV1 and FVC are within 10%, which indicates that the pulmonary function prediction by the combined algorithm has high accuracy among most testers and has the potential for wide application. However, there is an outlier in the FEV1 prediction, which indicates that there is still room for optimization of the combined algorithm prediction for individual testers. In terms of the overall distribution of errors, the combined algorithm achieved a good result for both FEV1 and FVC.

#### 3.3.3. Comparison of Algorithms

- Comparison of experimental results

To further demonstrate the superiority of this combination algorithm, we compared the results of the single-structure algorithm with the combination algorithm on the same data set, as shown in Table 7.

In Table 7, it can be seen that for the same pulmonary function parameter (FEV1 or FVC), the combined algorithm has the best prediction performance, followed by the traditional machine learning algorithm, and finally, the deep learning algorithm. The poor performance of the deep learning algorithm may be influenced by the small data set. Meanwhile, the processing method of the deep learning algorithm on the raw sequence information still needs further research. Traditional machine learning algorithms using medical features for pulmonary function parameter prediction are better overall but still need improvement in regard to MPE metrics. The combined algorithm performs optimally on this dataset, and all RMSE metrics less than 0.39 L. The R^2^ metrics are greater than 0.85, indicating that the predicted values are strongly correlated with the actual values. The combined learning algorithm incorporates medical feature information and sequence feature information to a certain extent, which can reduce the MAPE and MPE metrics of the predicted values and means that the prediction results have a greater application range.

- Comparison with state-of-the-art performance

As shown in Table 8, our results were further compared with the relevant literature. In terms of the number of people in the dataset, we used data from 1007 subjects, second only to the 3567 in [12]. With regard to the R^2^ index, this study obtained results greater than 0.85, which exceeds the results in the literature [11,13,14]. In terms of the RMSE metrics, our results also go beyond those in the literature [11,14]. In summary, the algorithm in this paper achieves high performance in the prediction of the relevant pulmonary function parameters.

## 4. Conclusions

In this paper, an algorithm combining traditional machine learning and deep learning was proposed to address the problem of the low accuracy of pulmonary function parameters in assessing respiratory diseases. The algorithm processes medical features by SVM and XGBoost algorithms to ensure the interpretability of the algorithm. The one-dimensional convolutional network is also used to analyze the CO_2_ series to fully explore the deep features in the sequence, and then the improved KNN algorithm is used to combine the results both simply and effectively to improve the accuracy of pulmonary function parameters. This algorithm can significantly improve the accuracy of pulmonary function parameter prediction in the assessment stage of respiratory diseases.

The proposed combined algorithm was compared with the single-structure algorithm and showed improvement in all regression metrics. The root mean square error (RMSE) of the pulmonary function parameters predicted by the combination algorithm was less than 0.39 L and the R^2^ was determined to be greater than 0.85 through a ten-fold cross-validation experiment. The algorithm was compared with other algorithms for pulmonary function parameter prediction, and the method was able to better utilize medical and serial features to achieve significant results. In addition, unlike most methods, the method proposed in this paper utilizes carbon dioxide volume data, which can be a better alternative to spirometry.

However, the algorithm proposed in this paper also has some limitations. The performance of the proposed algorithm needs to be improved when extracting information on sequence features. Additionally, this paper mainly focuses on the field of pulmonary function parameter prediction, and further research is needed to apply the algorithm to other fields. There are still some problems that need to be overcome in the course of further research. For example, the number of testers in this dataset is limited, so more samples are needed to validate the performance of the algorithm. Additionally, multidimensional test data can be incorporated for a more accurate prediction of pulmonary function parameters from multimodal data.

## Figures and Tables

**Table 1:** Configurations of sequence feature regression structure.

Layers (Type)	Output Size	Param
C1 (Conv1D)	(None, 2396, 32)	192
P1 (MaxPooling1D)	(None, 479, 32)	0
D1 (Dropout)	(None, 479, 32)	0
C2 (Conv1D)	(None, 475, 64)	10,304
P2 (MaxPooling1D)	(None, 95, 64)	0
D2 (Dropout)	(None, 95, 64)	0
C3 (Conv1D)	(None, 91, 32)	10,272
P3 (MaxPooling1D)	(None, 91, 32)	0
D3 (Dropout)	(None, 91, 32)	0
F1 (Flatten)	(None, 576)	0
F2 (Dense)	(None, 2)	1154

**Table 2:** The description of volumetric capnography features.

Variable	Description	Units
C12	Carbon dioxide concentration at the boundary of phase 1 and phase 2	mmHg
C23	Carbon dioxide concentration at the boundary of phase 2 and phase 3	mmHg
V12	Volume at the boundary of phase 1 and phase 2	mL
V23	Volume at the boundary of phase 2 and phase 3	mL
V2	The volume of phase 2	mL
V3	The volume of phase 3	mL
S2	Slope of phase 2	mmHg/L
S3	Slope of phase 2	mmHg/L
S3/S2	The ratio of slopes of phases 3 and 2	/
Angle23	The angle between phases 2 and 3	°

**Table 3:** Data description table.

	Amount	Category	Variable	Units	Values
Data	1007	Demographics	Male	%	53.1%
			Age	years	56 (14)
			Height	cm	166 (9)
			Weight	kg	69 (14)
			BMI	kg·m^−2^	24.94 (4.20)
		Volumetric capnography	C12	mmHg	2.49 (0.80)
			C23	mmHg	27.22 (4.76)
			V12	mL	276 (58)
			V23	mL	757 (157)
			V2	mL	480 (128)
			V3	ml	2061(903)
			S2	mmHg/L	74.63 (25.19)
			S3	mmHg/L	5.44 (3.37)
			S3/S2	/	0.08 (0.04)
			Angle23	°	168.26 (5.81)
		Spirometric	FEV1	l	2.53 (0.86)
			FVC	l	3.49 (0.99)

**Table 4:** Results of the conventional machine learning algorithm.

Type	Pulmonary Function Parameters	RMSE (L)	R^2^	ACC
SVM + XGBoost	FEV1	0.43	0.78	73.90%
	FVC	0.48	0.79	79.18%

**Table 5:** Results of the deep learning algorithm.

Type	Pulmonary Function Parameters	RMSE (L)	R^2^	ACC
1D-CNN	FEV1	0.66	0.57	65.09%
	FVC	0.61	0.73	74.76%

**Table 6:** Results of the combination algorithm.

Type	Pulmonary Function Parameters	RMSE (L)	R^2^	ACC
Combination algorithm	FEV1	0.35	0.85	80.79%
	FVC	0.39	0.86	85.77%

**Table 7:** Results of the different algorithms.

Parameter Types	Algorithm Types	RMSE (L)	R^2^(P)	MPE	MAPE	RMSPE	ACC
FEV1	SVM + XGBoost	0.43	0.78 (<0.01)	45.58%	15.71%	17.01%	73.90%
	1D-CNN	0.66	0.57 (0.02)	56.91%	21.51%	26.30%	65.09%
	combination algorithm	0.35	0.85 (<0.01)	32.84%	10.96%	13.83%	80.79%
FVC	SVM + XGBoost	0.48	0.79 (<0.01)	36.57%	12.26%	13.64%	79.18%
	1D-CNN	0.61	0.73 (<0.01)	44.30%	14.19%	17.22%	74.76%
	combination algorithm	0.39	0.86 (<0.01)	23.27%	8.35%	11.06%	85.77%

**Table 8:** Performance comparison with other works.

Author	Subjects	Methodology	Result
Sharan et al. [11]	322	Linear and nonlinear regression models	A root mean square error (and correlation coefficient) for standard spirometry parameters FEV1, FVC, and FEV1/FVC of 0.593 L (0.810), 0.725 L (0.749), and 0.164 L (0.547).
Ioachimescu et al. [12]	3567	Regular linear or optimized regression, ANN models	The AEX could become an essential tool in assessing respiratory impairment.
Miyoshi et al. [13]	683	Multivariate linear regression analysis	Actual and estimated VC, FVC, and FEV1 values showed significant correlations (all r > 0.8 and *p* < 0.001) in all groups.
Chen et al. [14]	143	M-SVR	The mean squared errors were lower than 0.15 l^2^, and the decision coefficients (R^2^) were higher than 0.40.
Ours	1007	SVM, XGBoost, 1D-CNN, KNN	The root mean squared errors (RMSE) were lower than 0.39 L. The coefficient of determinations (R^2^) was higher than 0.85. The comprehensive percentage error (CPE) was lower than 20%.

## Data Availability

Not applicable.
